# Cannabinomics: Application of Metabolomics in *Cannabis* (*Cannabis sativa* L.) Research and Development

**DOI:** 10.3389/fpls.2020.00554

**Published:** 2020-05-08

**Authors:** Konstantinos A. Aliferis, David Bernard-Perron

**Affiliations:** ^1^Laboratory of Pesticide Science, Agricultural University of Athens, Athens, Greece; ^2^Department of Plant Science, McGill University, Montreal, QC, Canada; ^3^The Green Organic Dutchman, Mississauga, ON, Canada

**Keywords:** cannabinoids, cannabis terpenoids, chemovars, drug discovery, medicinal cannabis, plant metabolomics, plant chemotaxonomy

## Abstract

*Cannabis* (*Cannabis sativa* L.) is a complex, polymorphic plant species, which produces a vast array of bioactive metabolites, the two major chemical groups being cannabinoids and terpenoids. Nonetheless, the psychoactive cannabinoid tetrahydrocannabinol (Δ*^9^*-THC) and the non-psychoactive cannabidiol (CBD), are the two major cannabinoids that have monopolized the research interest. Currently, more than 600 *Cannabis* varieties are commercially available, providing access to a multitude of potent extracts with complex compositions, whose genetics are largely inconclusive. Recently introduced legislation on *Cannabis* cultivation in many countries represents a great opportunity, but at the same time, a great challenge for *Cannabis* research and development (R&D) toward applications in the pharmaceutical, food, cosmetics, and agrochemical industries. Based on its versatility and unique capabilities in the deconvolution of the metabolite composition of complex matrices, metabolomics represents an ideal bioanalytical tool that could greatly assist and accelerate *Cannabis* R&D. Among others, *Cannabis* metabolomics or cannabinomics can be applied in the taxonomy of *Cannabis* varieties in chemovars, the research on the discovery and assessment of new *Cannabis*-based sources of bioactivity in medicine, the development of new food products, and the optimization of its cultivation, aiming for improvements in yield and potency. Although *Cannabis* research is still in its infancy, it is highly foreseen that the employment of advanced metabolomics will provide insights that could assist the sector to face the aforementioned challenges. Within this context, here, the current state-of-the-art and conceptual aspects of cannabinomics are presented.

## Introduction

**FIGURE 1:**
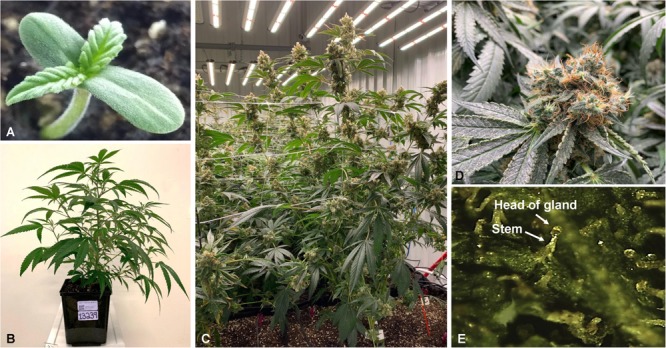
*Cannabis sativa* L.; One-week old seedling of the hemp dioecious strain “Finola” **(A)**, 4 weeks old plant of the strain “BIK” **(B)**, and plants at the flowering stage **(C)**. Close up photo of a flower of the strain “Skunk” **(D)**, and big capitate-sessile trichomes as shown in the stereomicroscope **(E)**.

*Cannabis* (*Cannabis sativa* L., Cannabaceae) (Figure 1) is a highly variable, complex, polymorphic plant species, which originates from Eurasia (Russo et al., 2008; Clarke and Merlin, 2013, 2016). Currently, it is distributed world-wide and grows in variable habitats, altitudes, and soil and climate conditions (Clarke and Merlin, 2016). There is a controversy among botanical taxonomists on the number of species that compose the *Cannabis* genus; presently, there is a consensus on the nomenclature proposed by Small and Cronquist (Small and Cronquist, 1976); *C. sativa* is monotypic, composed of two sub-species (subsp.), namely *sativa* and *indica*, based on their Δ*^9^*-tetrahydrocannabinol (Δ*^9^*-THC) content. The former is further sub-divided into two varieties (var.), *sativa* (low Δ*^9^*-THC, domestication traits) and *spontanea* (low Δ*^9^*-THC, wild-type traits), and the latter into var. *indica* (high THC, domestication traits) and var. *kafiristanica* (high Δ*^9^*-THC, wild-type traits). Approximately 600 *Cannabis* varieties are commercially available (Rahn et al., 2016), whose genetics, for many of these, are only partially known. The plant has a diploid genome (*2n* = 20) composed of nine autosomes and a pair of sex chromosomes (*X* and *Y*) (Ming et al., 2011) and its draft genome has recently been sequenced (Van Bakel et al., 2011).

**FIGURE 2:**
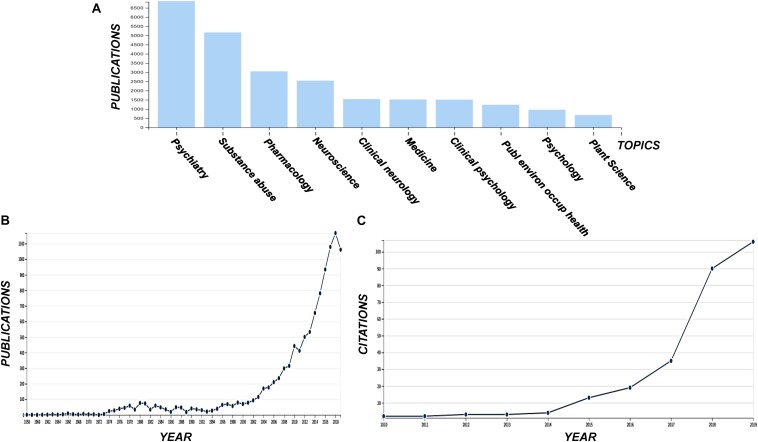
Publications grouped in various disciplines including the term “cannabis” **(A)** and the corresponding total number of publications **(B)**, and the number of citations acquiring for the terms “cannabis” and “metabolomics” **(C)**. Data were acquired from the data base of the ISI Web of Science (Clarivate Analytics, Philadelphia, PA, United States).

The use and exploitation of *Cannabis* has sparked controversy, however, the recent legalization of its use for medical and other purposes in many countries within the corresponding legislative framework (Pacula and Smart, 2017; Cox, 2018), in combination with the remarkable bioactivities of the plant, pose an urge for the acceleration and intensification of *Cannabis* research and development (R&D). Although it is still in its infancy, there is currently an exponentially increasing interest in *Cannabis* R&D, as it is confirmed by the number of relative publications and citations (Figure 2).

Nevertheless, drug discovery, the risk assessment of cannabis products and their quality control (QC), and the research on the plant and its bioactive constituents, necessitate the implementation of advanced bioanalytical tools. Such tools could facilitate the acquisition of the necessary missing knowledge that will be further exploited toward the development of innovative, safe products, and the improvement of the plant’s productivity in a timely fashion. Based on its versatility and unique capabilities in the deconvolution of the metabolite composition of complex matrices, metabolomics represents an ideal bioanalytical tool that could greatly accelerate *Cannabis* R&D. Its successful implementation requires solid expertise in experimental design, analytical and bioanalytical chemistry, advanced statistics, and bioinformatics. To date, metabolomics has been developed for a wide range applications in various fields such as plant (Sumner et al., 2015) and food science (Wishart, 2008; Cevallos-Cevallos et al., 2009; Herrero et al., 2012; Castro-Puyana and Herrero, 2013), medicine (Wishart, 2016), toxicology (Bonvallot et al., 2018; Viant et al., 2019), environmental sciences (Bundy et al., 2009), and plant protection products (PPPs) R&D (Aliferis and Chrysayi-Tokousbalides, 2011; Aliferis and Jabaji, 2011). Nonetheless, since comprehensive reviews on the topics of metabolomics methodologies, analytical platforms, software, and cannabinoid analysis have been recently published (Madsen et al., 2010; Aliferis and Chrysayi-Tokousbalides, 2011; Fuhrer and Zamboni, 2015; Gromski et al., 2015; Markley et al., 2017; Leghissa et al., 2018b; Pellati et al., 2018; Ramirez et al., 2019; Atapattu and Johnson, 2020), these topics are not reviewed here.

For the application of metabolomics in *Cannabis* R&D we are introducing the term “*Cannabinomics*” (Table 1). Its application could greatly assist the sector via the mapping of the metabolomes of the existing genotypes and their classification into the corresponding chemovars (Hazekamp et al., 2016; Lewis et al., 2018). Additionally, it has been predicted that the contribution of *Cannabinomics* toward the optimization and standardization of agricultural practices [e.g., application of plant growth regulators (PGR), bioelicitors, fertilizers, light conditions, irrigation events] for the production of superior quality products will be substantial (Magagnini et al., 2018). Similarly, it is expected to have a significant impact in the drug discovery, medicine, food science, functional cosmetics research, and metabolic engineering of microorganisms for the biosynthesis of cannabinoids. Here, the current state-of-the-art on these research topics, as well as conceptual aspects and perspectives, are being presented.

**TABLE 1:** Application of metabolomics in *Cannabis* research and development.

**Analytical method^a^**	**Extraction solvents^b^**	**Purpose of the study**	**References**
^1^H NMR	MeOH:H_2_O (1:1, v/v) or CHCl_3_-*d*, evaporation, dissolution in CHCl_3_-*d* or MeOH-*d*_4_:H_2_O-*d*_2_	Effect of jasmonic acid (JA) and pectin on *Cannabis* cell lines	Peč et al., 2010
^1^H NMR (^1^D DOSY) ^1^H NMR	H_2_O and H_2_O:EtOH extracts, evaporation, dissolution in CHCl_3_-*d*, MeOH-*d*_4_, or H_2_O-*d*_2_	Discovery of the differences among cultivars and study of the effects of temperature and solvent polarity on the cannabinoid content of extracts	Politi et al., 2008
^1^H NMR, ^1^H-^1^H COSY, ^1^H-^13^C HMBC	CHCl_3_-MeOH:H_2_O, evaporation of the extracts and finally dissolution in CHCl_3_-*d* or MeOH-*d*_4_:KH_2_PO_4_	Classification and analyses of *C. sativa* L. plants and cell suspension cultures	Flores-Sanchez et al., 2012
^1^H NMR	H_2_O-*d*_2_, CHCl_3_-*d*	Cannabinoids biosynthesis and metabolite profiles of trichomes during flowering	Happyana and Kayser, 2013
^1^H NMR LC/DAD	DMSO-*d*_6_ MeOH, MeOH:H_2_O	Discrimination among chemovars based on the cannabinoid and phenolic contents	Peschel and Politi, 2015
GC/FID	CHCl_3_, followed by Ace	Discrimination between *C. sativa* var *sativa* and *C. sativa* var *indica* based on the terpenoid profiles of essential oils	Hillig, 2004
GC/FID	EtOH	Chemotaxonomy of *Cannabis* strains based on their terpenoid and cannabinoid profiles	Fischedick et al., 2010
GC/FID	EtOH	Chemotaxonomy of *Cannabis* flower samples and extracts	Elzinga et al., 2015
GC/FID	EtOH	Chemotaxonomy of *Cannabis* strains based on their terpenoid and cannabinoid profiles	Hazekamp and Fischedick, 2012
GC/FID	EtOH	Chemotaxonomy of *Cannabis* strains based on their terpenoid and cannabinoid profiles	Hazekamp et al., 2016
GC/FID	MeOH	Chemotaxonomy of *Cannabis* strains based on their terpenoid profile	Fischedick, 2017
GC/FID, LC-DAD	EtOH	Method validation for the detection of cannabinoids and terpenoids	Giese et al., 2015
GC/FID, LC-DAD	MTBE	Chemotaxonomy of *Cannabis* strains based on their terpenoid and cannabinoid profiles	Zager et al., 2019
GC/MS	CHCl_3_, followed by evaporation of the extracts, and addition of Ace	Chemotaxonomy of *Cannabis* strains based on their Δ*^9^*-THC to CBD ratio	Hillig and Mahlberg, 2004
GC/MS	MeOH (80%, v/v)	Chemotaxonomy of *Cannabis* strains	Mudge et al., 2019
LC/ESI/MS	deionized H_2_O, followed by addition of ACN:MeOH 70:30 (v/v) (formic acid 0.1%, v/v), removal of phospholipids, drying, and dissolution in ammonium acetate (2.0 mM):ACN (70:30, v/v) solution	Study of pharmacokinetics of major cannabinoids in rat brains	Citti et al., 2018
LC/TOF/MS-LC/QTOF/MS	EtAc (formic acid 0.05% v/v).	Study and optimization of the biosynthesis of natural cannabinoids or synthetic analogs by metabolic engineered yeast strains	Luo et al., 2019
HRMS (Orbitrap MS)	MeOH	Chemotaxonomy of *Cannabis* strains and assessment of the quality of *Cannabis* products	Wang et al., 2018
LC/QQQ/MS NMR	MeOH, followed by dilution in H_2_O/MeOH (2/1, v/v) (0.1% formic acid) CHCl_3_-*d*	Analyses of plant’s trichomes	Happyana et al., 2013

*^*a*^^1^H-NMR; proton nuclear magnetic resonance spectroscopy, ^1^D DOSY; diffusion-edited ^1^H NMR, ^1^H-^1^H COSY; proton/proton correlation spectroscopy, ^1^H-^13^C HMBC; ^1^H-^13^C heteronuclear multiple quantum coherence, GC/FID; gas chromatography-flame ionization detector, GC/MS; GC/mass spectrometry, LC-DAD; liquid chromatography-diode array detector, LC/ESI/MS; liquid chromatography-electrospray ionization-mass spectrometry, LC/TOF/MS; liquid chromatography time-of-flight mass spectrometry, LC/QTOF/MS; quadrupole time-of-flight mass spectrometry, HRMS; high resolution mass spectrometry, LC/QQQ/MS; triple quadrupole LC/MS. ^*b*^Ace, acetone; CHCl_3_, chloroform; DMSO, dimethyl sulfoxide; EtAc, ethyl acetate; EtOH, ethanol; MeOH, methanol; MTBE, methyl tert-butyl ether.*

## Cannabis (*Cannabis Sativa* L.): A Unique Factory of Bioactive Metabolites and Multi-Complex Mixtures

**FIGURE 3:**
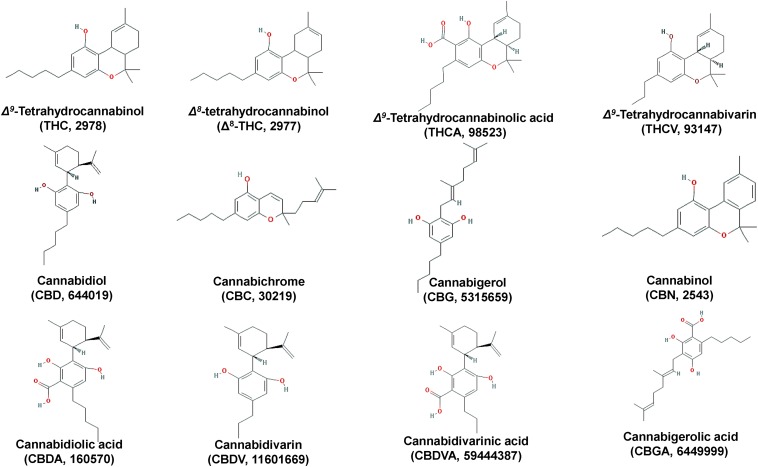
Chemical structures of major *Cannabis* (*Cannabis sativa* L.) cannabinoids.

**FIGURE 4:**
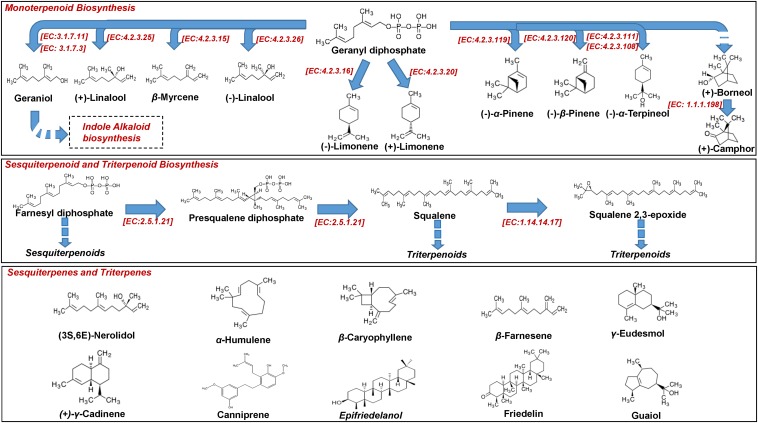
Biosynthesis of *Cannabis* (*Cannabis sativa* L.) mono-, sesqui, and triterpenoids.

**FIGURE 5:**
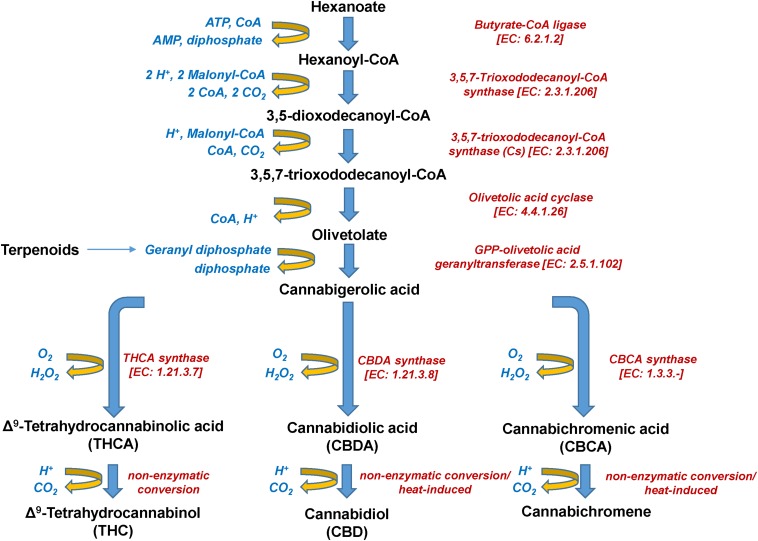
Biosynthetic pathway of *Cannabis* (*Cannabis sativa* L.) cannabinoids.

The plant owes its reputation to the biosynthesis of a vast array of diverse metabolites that exhibit unique structures, physicochemical properties, and bioactivities; cannabinoids, which is a unique class of secondary plant metabolites (Figures 3, 5) and terpenoids (Figure 4), are the most important groups of *Cannabis*-derived metabolites. To date, approximately 600 *Cannabis* metabolites have been isolated, with more than 20% of them belonging to cannabinoids (Chandra et al., 2017). Among them, seven have been classified as CBD-type metabolites (Morales et al., 2017). In addition to the bioactive metabolites, the plant is a rich source of cellulosic and woody fibers (Andre et al., 2016). Therefore, the discovery and functional characterization of all the genes involved in the biosyntheses of cannabinoids is of paramount importance for the development of various applications, as discussed below. Nonetheless, the application of metabolomics in the field is still in its infancy.

The psychoactive metabolite Δ*^9^-*THC and the non-psychoactive CBD (Figure 3), are the two major cannabinoids present in various concentrations in the different *Cannabis* chemovars, which largely determine their potency and pharmaceutical properties. The psychoactive and medicinal properties of *Cannabis* have been known for more than 5,000 years in the Middle East and Egypt, and later in China, India, Ancient Greece, and the Roman Empire (Di Marzo, 2008; Russo et al., 2008; Mechoulam and Parker, 2013; Farag and Kayser, 2015). Δ*^9^*-THC has monopolized the interest of the *Cannabis*-related R&D since its isolation in 1964 (Gaoni and Mechoulam, 1964) and total synthesis a year later (Mechoulam and Gaoni, 1965). On the other hand, CBD has recently attracted the interest of the scientific community mainly due to its, among others, antioxidant, anti-inflammatory, and analgesic properties (Morales et al., 2017). Based on such properties, it represents a model chemical structure of high potential in the synthesis of chemical analogs. In addition to Δ*^9^*-THC and CBD, other major cannabinoids are the cannabichromene (CBC), cannabidiolic acid (CBDA), cannabigerol (CBG), cannabinol (CBN), cannabidivarin (CBDV), cannabidivarinic acid (CBDVA), cannabigerolic acid (CBGA), cannabicyclol (CBL), Δ*^8^-*THC, tetrahydrocannabinolic acid (THCA), and tetrahydrocannabivarin (THCV) (Figures 3, 5).

A very interesting recent development is the biosynthesis of various cannabinoids by genetically engineered organisms, which could potentially provide solutions to the large-scale production of rare cannabinoids (Carvalho et al., 2017; Luo et al., 2019). The most profound example of such organism is yeast (*Saccharomyces cerevisiae*), which is a model that has been extensively used in metabolic engineering studies for the production of high-value chemicals (Liu et al., 2013; Nielsen et al., 2013; Carvalho et al., 2017). The biosynthesis of cannabinoids such as, CBGA, Δ*^9^*-tetrahydrocannabinolic acid, CBDA, Δ*^9^*-tetrahydrocannabivarinic acid, and CBDVA by metabolic engineered yeast strain has been recently reported (Luo et al., 2019). In this study, the carbohydrate galactose served as the precursor of cannabinoids, and to the best of our knowledge, this is the first report on the application of metabolite profiling applying liquid chromatography time-of-flight mass spectrometry (LC/TOF/MS)-quadrupole time-of-flight mass spectrometry (LC/QTOF/MS) analysis. The extraction was performed using ethyl acetate (EtAc-formic acid 0.05%, v/v). Within this context, as a functional genomics tool, metabolomics could ideally employed in the study and monitoring of the metabolism of engineered microorganisms toward the optimization of the biosynthesis of natural cannabinoids or their synthetic analogs.

Additionally, plants biosynthesize a vast array of lipophilic volatile metabolites via the removal of hydrophilic moieties in a series of reactions (e.g., reduction, methylation, acylation) (Pichersky et al., 2006). Such plant volatiles (PVs), among others, regulate their interactions with biotic and abiotic factors (e.g., attraction of pollinators, protection against pests and pathogens) (Dudareva et al., 2013). Among PVs, terpenoids represent the most important and populated chemical group, with the sub-groups of isoprenes (C_5_), monoterpenes (C_10_), and sesquiterpenes (C_15_) being the largest (Figure 4).

Terpenoids are synthesized via dimethylallyl diphosphate (DMAPP) and isopentenyl diphosphate (IPP) (Figure 4), which are derived from *Cannabis* biosynthetic pathways that are localized in different cell compartments (Nagegowda, 2010; Russo, 2011), sharing geranyl diphosphate (GPP) as a common precursor with cannabinoids (Grof, 2018). Playing a fundamental role in determining food’s flavor and fragrance, *Cannabis* terpenoids have recently attracted the interest of researchers (Russo and Marcu, 2017), threatening the dominance of Δ*^9^*-THC and CBD as its main potent metabolites. As presented below, the terpenoid profiles can be used in the classification of *Cannabis* chemovars (Fischedick, 2017) in addition to those of cannabinoids. The transcriptomics analysis of *Cannabis* trichomes has revealed that the plant is capable of synthesizing all of the known terpenes (Booth et al., 2017). In this study, transcripts that are associated with the biosynthesis of terpenes were found to be highly expressed in trichomes. Their biosynthesis is regulated by terpene synthases, which are organized in large gene families and their activity is spatially and temporally distributed, making them ideal targets for engineering (Tholl, 2006). Nonetheless, the biosynthetic pathway of terpenoids is highly complex, with recent studies highlighting the roles of novel genes that encode participating enzymes (Zager et al., 2019).

Terpenoids are highly potent metabolites, affecting the behavior of animals and even humans when inhaled at very low doses, and their synergy with cannabinoids has been proposed (Russo, 2011). Studies have highlighted the cornerstone role that cannabis mono- and sesquiterpenoids play in the potency of flower extracts and the “entourage effect” (Russo and Marcu, 2017). The in-depth understanding of the mechanism of the latter, although challenging, is highly anticipated to provide information that could be further exploited in various applications (e.g., medicine R&D). However, comparative study between terpenoid-rich essential oils and CBD confirmed the superior bioactivity and medicinal properties of the latter (Gallily et al., 2018). Terpenoids exhibited a transient immunosuppression and lower bioactivity levels (e.g., ROS scavenging properties) than CBD. In addition to their contribution to the properties of *Cannabis* extracts, individual terpenoids could be exploited *per se* as bioactive molecules (e.g., friedelin, canniprene, cannabisin, cannflavin A) (Russo and Marcu, 2017). For example, cannabisin B, which is isolated from the hempseed hull, has been found to induce autophagy human hepatoblastoma HepG2 cells (Chen et al., 2013).

## *Cannabinomics*: Applications of Metabolomics in *Cannabis* (*Cannabis Sativa* L.) Research and Development (R&D) and Current State-Of-The-Art

**FIGURE 6:**
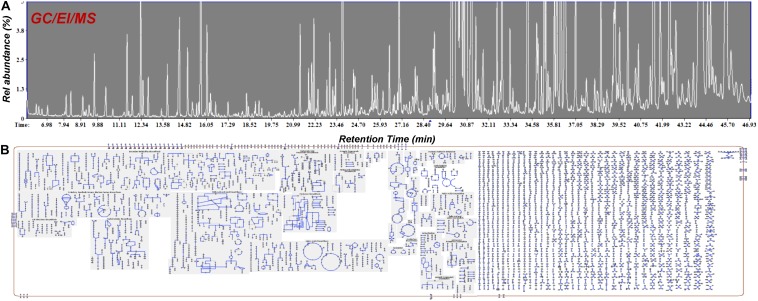
GC/EI/MS **(A)** total ion chromatograms of *Cannabis sativa* L. var Finola flower extracts. Approximately 220 metabolite features were discovered and **(B)** Cellular overview of the metabolite composition of *Cannabis* using the Plantcyc tools (Karp et al., 2009; Caspi et al., 2015).

Nuclear magnetic resonance (NMR) spectroscopy (Smolinska et al., 2012; Nagana Gowda and Raftery, 2016; Markley et al., 2017) and mass spectrometry (MS)-based (Hu et al., 2005; Dettmer et al., 2007; Ramautar et al., 2009; Fuhrer and Zamboni, 2015) analyzers are the two major analytical platforms employed in metabolomics analyses. Nonetheless, the integration of information on the metabolite composition of a certain sample that has been acquired by employing various analytical platforms is highly recommended, especially in the case of cannabis-derived matrices, which have highly complex metabolomes, composed of metabolites with highly diverse physicochemical properties (Figures 3–6; Andre et al., 2016).

In addition to the routine deconvolution of the composition of *Cannabis* flower and oil samples, there is an increasing interest on the analyses of the cannabinoid and terpenoid contents of a large array of diverse matrices such as, among others, edibles, medicine, cosmetics, blood, and urine, for research, regulatory, and law enforcement purposes (Jain and Singh, 2016; Meng et al., 2018). For the large-scale isolation of cannabinoid and terpenoid fractions or individual metabolites, the supercritical fluid extraction (SFE) and solid phase extraction (SPE) are the main employed methods (Rovetto and Aieta, 2017; Gallo-Molina et al., 2019). Nonetheless, for analytical and bioanalytical purposes, various extraction protocols have been proposed, with solid-based (e.g., solid-phase microextraction, SPME) and solvent-based (e.g., dispersive liquid-liquid microextraction, DLLME) ones being the preferred (Jain and Singh, 2016; Pellati et al., 2018; Ramirez et al., 2019; Atapattu and Johnson, 2020). Focusing on *Cannabis* metabolomics, the choice of the extraction protocol depends on the analytical platform and the aim of a given study (Table 1); in NMR analyses, chloroform (CHCl_3_)-*d*, methanol (MeOH)-*d_4_*, or H_2_O-*d*_2_ are the preferred solvents, ethanol (EtOH) for gas chromatography-flame ionization detector platform (GC/FID), MeOH for LC, and various solvents have been used in GC/MS-based studies. Further optimization of a given bioanalytical protocol (e.g., extraction, QC measures, analytical conditions, bioinformatics software) can lead to improved analytical capacities.

The capacity of NMR platforms in the recording of primary and secondary metabolites, and the integration of data acquired in various operating modes [e.g., proton NMR (^1^H-NMR), ^13^C-NMR, proton/proton correlation spectroscopy (^1^H-^1^H-COSY), heteronuclear multiple quantum coherence (HMQC), heteronuclear multiple bond correlation (HMBC)] for the structure elucidation of complex metabolites, represent major advantages in *Cannabis* R&D (Choi et al., 2004). The lyophilization is an important step in the pipeline of NMR analyses for the removal of water from the samples. NMR metabolomics has been applied in the classification and analyses of *C. sativa* L. plants and cell suspension cultures based on the recorded profiles of primary and secondary metabolites (Flores-Sanchez et al., 2012). In this study, following lyophilization, an indirect fractionation protocol was applied, which involves extraction of the dry plant material in a biphasic system (CHCl_3_-MeOH:H_2_O), evaporation of the extracts and finally dissolution in CHCl_3_-*d* or MeOH-*d*_4_:KH_2_PO_4_. A similar methodology has been applied in the study of the effects of jasmonic acid (JA) and pectin on two cell lines of *Cannabis*, which revealed a substantial impact of the treatments on the cells’ metabolism (Peč et al., 2010). In a first step, extraction of the lyophilized material was performed using MeOH:H_2_O (1:1, v/v) or CDCl_3_, followed by evaporation and dissolution in CHCl_3_-*d* or MeOH-*d*_4_: H_2_O-*d*_2_.

In another study, the potential of diffusion-edited (^1^D DOSY) ^1^H NMR metabolomics in the assessment and optimization of extraction protocols was investigated (Politi et al., 2008). The developed protocol enabled the recording of metabolite profiles of H_2_O and H_2_O:EtOH extracts that could be used to discover differences among cultivars and the effects of parameters, such as temperature and solvent polarity on the cannabinoid content of extracts. Furthermore, ^1^H NMR, using deuterated dimethyl sulfoxide (DMSO-*d*_6_) as the extraction solvent, has a proven capacity and potential in the high-throughput discrimination between *Cannabis* chemovars, following chemotaxonomy approaches. Its integration with liquid chromatography-diode array detector (LC/DAD) analyses has enabled the discrimination among four chemovars based on their cannabinoid and phenolic contents (Peschel and Politi, 2015).

Cannabinoids can be analyzed by employing both GC- and LC-based analyzers (Giese et al., 2015; Leghissa et al., 2018b). However, issues with their conversion under the high temperatures of the injection port of the former, make their absolute quantification tricky, and their analyses preferable by using LC-based analyzers. On the other hand, although terpenoids can be recorded by EI detectors, their structural similarities make their absolute identification challenging. Thus, GC/FID platforms are suitable for the analyses of terpenoid profiles (Giese et al., 2015; Leghissa et al., 2018b). Additionally, the linear range of the detector facilitates the recording of the wide range of terpene concentrations in *Cannabis* extracts. The aforementioned, make its employment important in the recording of terpenoid profiles and the assessment of the bioactivity and potency of the analyzed samples.

Furthermore, analyzers equipped with triple quadrupole (QQQ) detectors such as LC/QQQ/MS and GC/QQQ/MS systems, are very important in *Cannabis* research due to their superior selectivity and sensitivity in quantitative analyses (Leghissa et al., 2018b; Ramirez et al., 2019). The ability to operate these detectors in different modes such as, multiple reaction monitoring (MRM) or selected reaction monitoring (SRM), represents an advantage for *Cannabis* metabolomics. MRM is the most commonly employed method for the quantification and identification of metabolite features, owning its potential to the sensitivity, linear dynamic range, and specificity (Leghissa et al., 2018a). However, their performance declines during the analyses of large numbers of metabolites. Such disadvantage could be addressed by the employment of time-of-flight analyzers (ToF), which offer superior mass resolution and accuracy, facilitating fast scan speeds and enable the deconvolution of overlapping analytes (Beale et al., 2018). Furthermore, two-dimensional gas chromatography (GC × GC) systems could improve the separation of co-eluting metabolites (Mondello et al., 2008; Beale et al., 2018) and improve our capacities in deconvoluting complex *Cannabis*-derived matrices.

Interestingly, during the injection of cannabinoid-containing samples in GC-based systems, their acidic forms (e.g., THCA, CBDA, CBCA) entirely convert (decarboxylation) to their neutral products (e.g., Δ*^9^*-THC, CBD, CBC) (Figure 5). This is probably the result of the high temperatures being applied in the injector, which commonly exceed 260°C. Although EI coupled with GC/MS analyzers seems to be more efficient than APCI or ESI in cannabinoid analysis due to the improved fragmentation (Leghissa et al., 2018b), the observed conversions could possibly result in the recording of false-positives for Δ*^9^*-THC, CBD, or CBC. This, in turn, jeopardizes analyses, posing serious risks toward the successful QC and the validity of research results. Such conversions can be avoided by appropriate silylation of the analyzed samples (Leghissa et al., 2018a) and further measures such as the use of isotopically-labeled standards, could greatly improve the accuracy of analyses.

For QC purposes, the implementation of different analyzers is required for the monitoring of metabolites across the various groups of *Cannabis* metabolites, which exhibit highly diverse physicochemical properties, making their detection and quantification challenging tasks. The employment of LC-diode array detector (LC-DAD) and GC/FID platforms have enabled the repeatable detection of cannabinoids and terpenes with low relative standard deviations (RSDs), using EtOH for extraction (Giese et al., 2015).

Additionally, high-resolution mass spectrometry (HRMS) [e.g., Fourier-transform ion cyclotron resonance (FT-ICR)-MS, Orbitrap analyzers] represents one of the latest developments in analytics. Commonly hyphened with LC, HRMS analyzers facilitate the coverage of a larger portion of the metabolite composition of the analyzed samples than that achieved by the conventional analyzers. Although optimization of the analytical conditions is required (e.g., binning, resolving powers), HRMS has a great potential in the chemotaxonomy of *Cannabis* chemovars and the assessment of the quality of *Cannabis* products (e.g., potency, authentication) (Wang et al., 2018).

### Dissection of the Cannabinoid Biosynthesis by the Glandular Trichomes

Cannabinoids naturally occur in plants in the acidic form, with their corresponding decarboxylated analogs being the result of non-enzymatic catalyzed reactions during their storage or heating (Figure 5). The olivetolic acid cyclase (OAC, EC 4.4.1.26) is a unique type III polyketide synthase (PKS) and key enzyme in the cannabinoid biosynthetic pathway (Morita et al., 2019) together with a tetraketide synthase (*C. sativa* TKS; CsTKS) (Taura et al., 2009). OAC is a dimeric α + β barrel (DABB) protein, which exhibits structural similarities to polyketide cyclases of *Streptomyces* sp. (Gagne et al., 2012). Interestingly, it is the only known plant polyketide cyclase that can accept directly a linear poly-β-ketide intermediate, which is required for the biosynthesis of olivetolic acid (OA) (Marks et al., 2009; Gagne et al., 2012; Morita et al., 2019). The enzyme is over-expressed in the glandular trichomes (Gagne et al., 2012) and its structure has been recently studied (Yang et al., 2016). OA, in turn, forms the polyketide nucleus of cannabinoids (Figure 5). The precursor of cannabinoids hexanoyl-CoA, has been primarily detected in female *Cannabis* flowers by employing LC-MS/MS, with lower amounts recorded in the leaves, stems, and roots (Stout et al., 2012). Such pattern follows the accumulation of the end-products of cannabinoids. Hexanoyl-CoA can be synthesized via the *de novo* biosynthesis of fatty acids or the breakdown of lipids. Nonetheless, the potential of metabolomics in the dissection of PKS and the discovery of the functional links between the *Cannabis* genome, transcriptome, and metabolome is largely unexploited.

The plant has a variety of non-glandular and glandular trichomes on its flowers, which are the production sites of phytochemicals; the biosyntheses and accumulation of cannabinoids and essential oils take place in the glandular trichomes, where a terpene-rich resin is produced (Figure 1E). Three types of glandular trichomes occur in *Cannabis*; capitate-stalked (Figure 1E), capitate-sessile, and bulbous trichomes. The development of the secretory cavities and the fine structure of trichomes have been thoroughly examined in the course of flowering by transmission electron microscopy (TEM) (Kim and Mahlberg, 1991) and scanning electron microscopy (SEM) (Happyana et al., 2013). There are two major groups of glandular trichomes, the first includes those with glands whose heads are composed of eight cells and the second, glands whose heads are usually composed of two cells, with a maximum of four (Dayanandan and Kaufman, 1976).

The superior capacity of metabolomics in the deconvolution of complex matrices is a major advantage in the study of the biosynthesis of cannabinoids by the glandular trichomes of the plant. ^1^H NMR-based metabolomics combined with real-time PCR analyses have been employed in the study of the metabolite profiles of the trichomes of the *C. sativa* varieties Bediol, Bedica, Bedrobinol, and Bedrocan, during the last 4 weeks of their flowering (Happyana and Kayser, 2013). In the chloroform extracts, the cannabinoids Δ^9^-THC, THCA, CBD, CBDA, and CBCA were identified, whereas in the water extracts, several amino acids, carbohydrates, and various other metabolites were detected. The similar fluctuations of the levels of cannabinoids with those of the corresponding encoding genes suggested a decline in the cannabinoid biosynthesis of the plant near the end of the flowering period. THCA and CBDA were discovered as the cannabinoids with the highest leverage in the observed fluctuation of the metabolite profiles of the trichomes. LC/QQQ/MS (solvent; MeOH) and NMR analyses (solvent; CHCl_3_-*d*) have also revealed the presence of several major as well as minor cannabinoids in the plant’s trichomes, which further confirm their importance and role in their biosynthesis (Happyana et al., 2013). The employment of these two analyzers following the developed analytical protocols resulted in the detection of the acidic forms of the metabolites, with only minor quantities of their corresponding forms detected.

Such studies highlight the potential of metabolomics in the determination of the optimal time of harvesting of a given strain under specified conditions in order to improve the yield and quality of the obtained products.

### Chemotaxonomy of Varieties: Chemovars

The domestication of *Cannabis* and the, until recently, illegal status of its cultivation, have resulted in a vast number of genotypes, which exhibit largely unknown properties and genotypic and metabolic backgrounds (Mudge et al., 2018). Although from a botanical perspective, the conventional taxonomy classification system is relevant, focusing on *Cannabis*, the taxonomy of its strains based on their content in potent metabolites (e.g., cannabinoids, terpenoids) in the so-called chemovars, seems to be the most appropriate for R&D purposes. A data survey suggests that there has been a steady trend in favor of higher Δ*^9^*-THC content in herbal and resin samples; from 13 to 23% in mid-2016, compared to 7–10% in 2009. That indicates a biased selection in favor of high potency chemovars of medicinal *Cannabis* (Dujourdy and Besacier, 2017). The differentiation between chemovars in their cannabinoid content is explained by the differences in the expression of genes that encode their biosyntheses (Van Bakel et al., 2011). To date, GC/FID platforms have been mainly employed in chemotaxonomy studies on *Cannabis*.

*Cannabis* strains are grouped in three types, Type I (high Δ*^9^*-THC content), Type II (various Δ*^9^*-THC to CBC ratios), and Type III (high CBD content) (Lewis et al., 2018). However, since additional *Cannabis* metabolites are bioactive, with a major group being the terpenoids, the classification of chemovars that takes into account the sum of its bioactive components has also been proposed (Hazekamp and Fischedick, 2012; Hazekamp et al., 2016; Fischedick, 2017), and probably best describes their properties.

Employing a GC/FID platform for the chemotaxonomy of high Δ*^9^*-THC-producing *Cannabis* strains, and using MeOH as the extraction solvent (Fischedick, 2017), the application of multivariate analysis enabled their grouping into 13 chemovars based on their terpenoid profiles. GC/MS has been employed in the classification of *C. sativa* var *sativa* or *C. sativa* var *indica* strains based on their Δ*^9^*-THC to CBD ratio (Hillig and Mahlberg, 2004). Samples were extracted in CHCl_3_, followed by evaporation of the extracts and finally, addition of acetone (Ace). Most chemovars with Δ*^9^*-THC/CBD ratio greater than 25% were grouped as *C. sativa* var *indica*, while those with a ratio lower than 25% as *C. sativa* var *sativa*. Additionally, there was a high correlation between the content of chemovars in tetrahydrocannabivarin (THCV) and cannabidivarin (CBDV) and their grouping as *C. sativa* var *indica*.

Additionally, the terpenoid profiles can be used in the chemotaxonomy of the various *Cannabis* chemovars. Plants of diverse genetic backgrounds of *C. sativa* var *sativa* and *C. sativa* var *indica*, can be discriminated based on the terpenoid profiles of their essential oils using a GC/FID platform (Hillig, 2004). Plant material was extracted in CHCl_3_, followed by extraction in Ace. Employing the same analyzer, *Cannabis* terpenoids and cannabinoids following the extraction of plant material with EtOH, were quantitatively analyzed for the classification of 11 strains into chemovars (Fischedick et al., 2010). The profiling based on 36 compounds was successful in discriminating the varieties applying multivariate analysis. Based on a similar bioanalytical protocol, employing LC-DAD and GC/FID analyzers, nine strains of commercial *Cannabis* were grouped in *C. indica*-dominant and *C. sativa*-dominant, based on their cannabinoid and terpenoid contents (Zager et al., 2019). The plant tissues were extracted using methyl tert-butyl ether and 1-octanol as the internal standard. Results of metabolite profiling were combined with results of RNA-seq for the transcriptome of the glandular trichomes. Interestingly, the study revealed similar patterns between the fluctuations of metabolite and transcript levels. Such observation confirms the applicability and potential of metabolomics in multi-level omics studies toward the understanding of the metabolism regulation, which is crucial in *Cannabis* research. A GC/FID analyzer and multivariate analysis were also employed in the discrimination of a large number of *Cannabis* flower samples and extracts into chemovars based on the analysis of their EtOH extracts (Elzinga et al., 2015). The analyzed strains exhibited variable reproducibility in the obtained metabolite profiles, with several terpenoids serving as biomarkers for the discrimination between the analyzed strains. Interestingly, it was also discovered that although quantitatively different, the chemical profiles of flowers and those of the extracts were qualitatively similar. Following a similar bioanalytical protocol using EtOH as the extraction solvent, 28 monoterpenoids, sesquiterpenoids, and cannabinoids were used for the classification of commercial *Cannabis* strains in various chemovars and the assessment of their quality (Hazekamp and Fischedick, 2012). The same research group has successfully analyzed 460 *Cannabis* accessions by GC/FID, aiming in their classification as “*sativa*” or “*indica*” based on their cannabinoid and terpenoid contents (Hazekamp et al., 2016). The extraction was performed using EtOH and 1-octanol served as the internal standard. The chemotaxonomy of *Cannabis* in chemovars based on their terpenoid profiles has also been performed by headspace GC/MS analysis, using MeOH (80%, v/v) for the extraction (Mudge et al., 2019). The applied protocol enabled the grouping of the analyzed strains in 33 chemovars, with their content in the sesquiterpene caryophyllene oxide to be strongly correlated with high Δ*^9^*-THC content. LC hyphened to UV detectors has also been employed in the rapid grouping of strains in chemovars based on their content in major cannabinoids (Mudge et al., 2016).

### *Cannabis* as a Source of Novel and Unique Bioactive Compounds

There is no doubt that *Cannabis* with the chemical diversity, unique structures (Figures 3, 4), physicochemical properties, and diverse bioactivities of its metabolites, represents an invaluable source for the development of novel applications in various sectors, such as, medicine, cosmetics, and the food industry. Although such applications are yet in their infancy, it is anticipated that *Cannabis*-based or *Cannabis*-infused products will provide solutions to major human health conditions, and lead to the development of new functional food and beverage products.

Nonetheless, the complexity of the plant’s extracts and the in-depth understanding of interactions between their components (e.g., entourage effect) and synergism, represent major challenges. The development of pharmaceuticals based on *Cannabis* extracts is challenging for the medicinal research, which operates according to the principle “single compound-single target” (Hazekamp et al., 2016). However, the ineffectiveness of individual compound-based medicine against multigenic diseases (e.g., cancer) or diseases that affect multiple tissues dictate the need for the discovery of drugs that will act on multiple targets (Zimmermann et al., 2007; Giordano and Petrelli, 2008). Therefore, it is of paramount importance to distinguish between the bioactivities of mixtures and those of the individual bioactive metabolites based on appropriate protocols, which could be greatly assisted by high-throughput metabolomics. Examples of *Cannabis*-derived pharmaceuticals are displayed in the Table 2.

**TABLE 2:** Examples of *Cannabis*-derived pharmaceuticals.

**Name**	**Active ingredients (a.i.)**	**Indications**
Bedrocan^®^ Cannabis flos (dry flower from various cultivars) or granules	Standardized, consistent composition of cannabinoids and terpenes	• Pain, spasms and inflammation, often associated with MS • Chronic nerve pain.
Cannador^®^	THC:CBD ratio approximately 2:1	• Clinically tested for reduction of muscle stiffness, spasms and pain in Multiple Sclerosis • Annorexia/cachexia in cancer patients • Post-operative pain management.
Dronabinol (Marinol^®^, Syndros^®^)	Δ^9^-Tetrahydrocannabinol (Δ^9^-THC) (synthetic cannabinoid)	• Nausea and vomiting associated with cancer chemotherapy • Loss of appetite and weight loss in people with HIV infection • Sleep apnea reliever
Nabilone (Cesamet^®^, Canemes^®^)	Nabilone (synthetic cannabinoid)	• Nausea and vomiting associated with cancer chemotherapy
Sativex^®^	Δ^9^-THC 27 mg mL^–1^ (from Tetranabinex – *Cannabis sativa* L. extract) cannabidiol (CBD) 25 mg mL^–1^ (from Nabidiolex – *C. sativa* L. extract)	• Treatment for the symptomatic relief of neuropathic pain in multiple sclerosis (MS) in adults

Within this context, the discovery, assessment, and development of new sources of bioactivity as drugs for the treatment of various conditions, represent key priorities for the medicinal R&D (Chin et al., 2006; Dittrich and Manz, 2006; Harvey, 2008). Based on results of recent research, there is a growing amount of evidence that supports the effectiveness of various *Cannabis*-derived cannabinoids in the treatment of a wide range of conditions, including, among others, chronic and acute pain, epilepsy, sleep disorders, multiple sclerosis, gastrointestinal reflux disease, irritable bowel syndrome (IBS), spasticity, hypertension, and schizophrenia (Pacher et al., 2005; Hazekamp and Grotenhermen, 2010; Caraceni et al., 2014; Bruni et al., 2018).

The psychoactive and medicinal properties of *Cannabis* have been known for more than 5 millennia by major civilizations of the Middle East, Egypt, China, India, Ancient Greece, and the Romaine Empire (Di Marzo, 2008; Mechoulam and Parker, 2013; Farag and Kayser, 2015). Cannabinoids were the first identified group of potent *Cannabis* metabolites, with the medicinal properties of its major representatives being attributed to their interference with the G protein-coupled cannabinoid receptors (GPCRs) CB1 and CB2 of the endocannabinoid system (Di Marzo et al., 2004; Mechoulam and Parker, 2013). The CB1 receptors are amongst the most abundant GPCRs in the brain of mammals and are also present, to a lesser extent, in various peripheral organs, whereas the CB2 receptors have been identified throughout the central nervous system (CNS) and cells of the immune system, being part of a general protective system (Di Marzo et al., 2004; Di Marzo, 2008; Mechoulam and Parker, 2013) and modulating cytokine release (Pertwee, 2005).

Although Δ*^9^*-THC was isolated and synthesized in the mid 60s’ (Gaoni and Mechoulam, 1964; Mechoulam and Gaoni, 1965), the research on the mode(s)-of-action (MoA) of cannabinoids remained inconclusive for more than 20 years (Mechoulam and Parker, 2013). The similarities between the physicochemical properties and structures of cannabinoids (Figure 3), pose an obstacle toward their isolation in pure chemical form (Mechoulam and Hanuš, 2000) and the subsequent investigation of their bioactivities, MoA, and pharmacokinetics.

Furthermore, the cannabinoid interconversions during storage and heating are complex (Figure 5), which represents a major challenge for the development of new *Cannabis*-based products, such as drugs, cosmetics, beverages, and edibles. Additionally, of great interest is the fact that non-psychoactive *Cannabis* metabolites (e.g., terpenoids) can act synergistically with Δ*^9^*-THC, contributing to the so-called “entourage effect” of medicinal *Cannabis* extracts (Ben-Shabat et al., 1998; Russo, 2011, 2018), with the undergoing operating mechanism(s) being largely unexplored.

Another major challenge for the *Cannabis* industry related to drug development is the production of standardized extracts that will meet the standards set by the corresponding regulatory agencies (e.g., Cannabis Act, Canada)^1^. The agricultural practices, plant growth conditions, and extraction processes all play key roles in achieving consistency of the extracts’ content, however, discussion on those factors are beyond the aim of the present review. The robust QC of *Cannabis* preparations and assessment of their consistency and potency could be achieved by applying metabolomics for the various batches of a given product. The application of metabolomics employing and integrating information acquired by various analyzers (e.g., LC and GC-based platforms) could lead to the deconvolution of the complex chemical composition of *Cannabis* extracts and the monitoring of the consistency across batches, facilities, and different cultivation periods. For R&D purposes, metabolomics could be employed in the optimization of agricultural practices, growth conditions, and extraction processes in order to achieve the desired composition of extracts with proven medicinal properties, as discussed below. To the best to our knowledge, such approach is in its infancy, and no reports are currently available.

Cannabinoids exert palliative effects in cancer patients by, among others, preventing nausea and pain, and stimulating appetite (Guzman, 2003). Additionally, it has been shown that they inhibit the growth of tumor cells *in vitro* and *in vivo* in animal models (Guzman, 2003) and exhibit antitumor activity (Velasco et al., 2012; Dando et al., 2013). Such bioactivities have been supported by Phase III clinical trials, however, the corresponding mechanism(s) of action remain inconclusive. In the case of pancreatic adenocarcinoma it seems that cannabinoids induce autophagy and inhibit cell growth (Dando et al., 2013).

CBD, the second-most studied cannabinoid, and various of its synthetic derivatives have attracted the interest of the pharmaceutical industry and that of academic researchers, with specific focus on the understanding of their MoA, potency, and pharmacokinetics (Morales et al., 2017). It exhibits remarkable potency, including sedative, anxiolytic, anticonvulsive, hypnotic, anti-psychotic, anti-nausea, and anti-inflammatory effects (Mechoulam et al., 2002). Preclinical studies have highlighted the inflammatory potential of CBD in mouse models (Morales et al., 2017), without causing behavioral changes (Viudez-Martínez et al., 2018). It exerts a well-documented anti-seizure and anti-epileptogenic properties against epilepsy independent of the CB1/CB2R, which is supported by Phase III clinical trials on treatment-resistant epilepsies (Rosenberg et al., 2017). Additionally, information on the action of Δ*^9^-*THC containing Cannabis preparations in the treatment of pediatric epilepsies remains largely fragmented (Rosenberg et al., 2017). On the other hand, Δ*^9^-*THC or synthetic cannabinoid-induced seizures in mice have been observed following their intraperitoneal administration, which can be prevented by a CB1-selective antagonist (Malyshevskaya et al., 2017).

In addition to the two major cannabinoids Δ*^9^*-THC and CBD, other cannabinoids with limited or no psychoactive properties could exhibit interesting pharmaceutical properties and bioactivities. Among those are cannabidiol and cannabinoic acids, whose MoA are yet unknown (Di Marzo et al., 2004). Several cannabinoids (e.g., Δ*^9^*-THC, CBD, CBC, CBG, CBN), exhibit antibiotic activity to *Staphylococcus aureus*, highly correlated to the stereochemistry of the molecules and the groups of substitution (Appendino et al., 2008).

Additionally, the biotransformation of cannabinoids in the human body, which determines their potency and medicinal properties, is a largely unexplored topic and could lead to the discovery of novel bioactive metabolites (Dinis-Oliveira, 2016). Due to their high lipophilicity, cannabinoids could remain in the plasma and fat tissue for prolonged periods. Focusing on the Δ*^9^*-THC, in a first phase (Phase I, oxidative metabolism), it is metabolized to *11*-hydroxy-Δ*^9^*-THC, which is further metabolized to the inactive *11*-nor-*9*-carboxy-Δ*^9^*-THC. The Phase II (conjugation metabolism), includes reactions such as conjugation which lead to the detoxification of the molecule (Dinis-Oliveira, 2016). More than 80 Δ*^9^*-THC-derived metabolites have been identified as products of its transformation (Mazur et al., 2009).

The integration of information from clinical trials in which patients provide feedback following treatments with various *Cannabis* chemovars and information on the corresponding metabolite profiles employing metabolomics is very important for the selection of the best varieties and their standardization for medical use and drug discovery purposes. Based on this approach, applying GC/FID/MS metabolomics, Dutch researchers evaluated 460 accessions based on their content in major cannabinoids and terpenes (Hazekamp et al., 2016). Results revealed a strong correlation between *Cannabis* phenotypes and their terpene content, as it can be evaluated by their smell, taste, and medicinal properties, as well the importance of gibberellic acid (GAs) in terpenoid biosynthesis.

LC/ESI/MS-based metabolomics has been employed in the study of pharmacokinetics of major cannabinoids in rat brains, following their oral administration (Citti et al., 2018). Brains were initially homogenized in deionized H_2_O, followed by the addition of ACN:MeOH 70:30 (v/v) containing formic acid (0.1%, v/v). Following the removal of phospholipids, the extracts were dried and finally an ammonium acetate (2.0 mM):acetonitrile (70:30, v/v) solution was added. Analysis revealed the formation of novel, unique CBD-derived metabolites and fluctuations in the levels of several other endogenous metabolites as a result. Such application confirms the potential of metabolomics in the acquisition of fundamental knowledge related to the study of the mode(s)-of-action and bioactivity of cannabinoids for medical purposes.

The function of the endocannabinoid system and its regulation by endocannabinoids are complex, and yet relative information is largely fragmented. Their levels and relative composition vary depending and their role, which could shift from protective to deregulator of the physiological state of an individual. Therefore, compounds that could prolong the lifespan or suppress endocannabinoids could be extremely important in treating various conditions (Di Marzo, 2008).

### *Cannabis* in the Food Industry: Exploring the Potential and Assessing the Associated Risks

Canada (Federal level) (Cox, 2018), the United States of America (Individual States) (Pacula and Smart, 2017), and Uruguay, have pioneered the legislation on *Cannabis* use for medicinal and recreational purposes. In contrast to the research on the plant as a source of bioactivity for applications in medicine as described above, the corresponding research on its use as a food ingredient is in its first steps (Charlebois et al., 2018). A wide variety of methods exist for consuming *Cannabis* edibles for medical purposes such as, concentrated oils, tinctures, and oil capsules, whereas from a recreational perspective, edibles could be considered cannabis-infused food products and beverages, with the latter being less popular (Blake and Nahtigal, 2019).

Food metabolomics, or foodomics, has established itself as a robust and precise bioanalytical tool in the assessment of quality and safety of raw materials and food products, as well as in the assessment and optimization of processing protocols and procedures (Wishart, 2008; Cevallos-Cevallos et al., 2009; Herrero et al., 2012; Castro-Puyana and Herrero, 2013). MS-based analytical platforms hyphened with various detectors and NMR have been employed in food research and also the routine QC of food products (Cevallos-Cevallos et al., 2009; Ibáñez et al., 2013). Food samples could be solid, semi-solid or liquid, and they are composed of a vast number of compounds such as, small molecular weight metabolites (e.g., amino acids, carbohydrates, carboxylic acids, fatty acids), proteins, and peptides, thus, generating very complex matrices. In the case of *Cannabis*, the presence of a large number of lipophilic cannabinoids and terpenoids, together with primary and secondary metabolites, results in one of the most challenging matrices to be analyzed (Figure 6). Therefore, the analyses of cannabis-infused food becomes extremely challenging, requiring the implementation and integration of advanced analyzers.

Nevertheless, the application of advanced metabolomics in the monitoring of the global metabolite profiles of *Cannabis*-infused edibles and beverages could provide valuable insights into the stability of cannabinoids and other Cannabis-derived metabolites in the food matrices, their fate and interconversions during processing, and possible toxicity issues. Additionally, it could reveal the links between their organoleptic and medicinal properties, and potency with their metabolite composition, that could be further exploited in drug discovery and the development of new food products. Nonetheless, the task of developing validated protocols for the analyses of a large array of *Cannabis* metabolites in food matrices is challenging, and currently, only a few relative studies have been published (Escrivá et al., 2017; Meng et al., 2018). Although THC-infused food could spark public and scientific controversy, the fact that CBD exhibits interesting bioactivities, while at the same time being non-psychoactive, possibly makes it a promising candidate for the large-scale production of functional CBD-infused edibles or beverages. However, since research in the field in its first steps, the use of cannabinoids in food should undergo thorough research and assessment prior to the commercialization of related products.

### Regulation of *Cannabis* Metabolism Toward the Optimization of the Yield and the Biosynthesis of Bioactive Products

#### Effect of Light Conditions on *Cannabis* Growth: Phenotypes and Metabolomes

As it is the case with all plant species, the light regime is an important growth factor in *Cannabis* cultivation, being a fundamental component for the optimization of every successful growth protocol. The intensity, quality, and duration of light are among the most important factors that regulate plants’ physiology, development, and morphogenesis (Burgie et al., 2014; Galvão and Fankhauser, 2015; Krahmer et al., 2018). For the processing of the information relative to light regimes, plants are equipped with a series of photoreceptors capable of sensing a broad light spectrum (280–750 nm, UV-B to far-red) that are present in all of their compartments (Kami et al., 2010; Galvão and Fankhauser, 2015). Based on research using *Arabidopsis* as the model organism, it has been discovered that the phytochromes A-E (PhyA-PhyE) are responsible for sensing the red (R) and the far-red (FR) light, three classes of photoreceptors were assigned as sensors of the UV-A/blue light, whereas data on UV-B were inconclusive (Kami et al., 2010). An early study on *Cannabis*, has indicated a linear increase in the Δ*^9^*-THC content of leaves and flowers of medicinal chemovars with the UV-B irradiation level (Lydon et al., 1987). However, treatments had no effect on the levels of other cannabinoids in both the medicinal and industrial chemovars being studied.

In *Cannabis* research, among others, the in-depth understanding of its transition to the flowering stage is of great importance. Light as well as temperature, regulate the transition to the reproductive growth through their effects on the complex regulatory plant metabolic networks (Kami et al., 2010). Although evidence offers some understanding on the roles of phytochromes in plants’ development and morphogenesis, information on the correlation between their function and the regulation of plants’ primary and secondary metabolism is still largely fragmented. The acquisition of such knowledge represents a challenge but at the same time a great opportunity for *Cannabis* metabolomics. Furthermore, the recent developments related to the study of the effects of light on plants have been tremendous since the introduction of light-emitting diodes (LEDs), which are replacing the gas-discharge lamps. National Aeronautics and Space Administration (NASA) researchers discovered LEDs in their effort to grow plants in space (Stutte, 2015). LED technology enables a vast variety of light regimes to be applied on plants in order to regulate photosynthesis, morphogenesis, and growth according to our needs, with a low thermal energy output.

Experiments with tomato (*Solanum lycopersicum* L.) have shown that the blue and purple lights reduce photosynthesis, enhance the cyclic electron flow (CEF) and induce energy dissipation for photoprotection of the photosystems I and II (PSI and PSII, respectively) (Yang et al., 2018). The exposure of plants in different intensities of monochromatic red-LED affect their central metabolism and the size of the fruits produced (Fukushima et al., 2018). Additionally, LEDs have been reported to affect the reactive oxygen species (ROS) redox, antioxidant responses, and the *in vitro* regeneration of plants (Gupta and Agarwal, 2017).

The urge to improve *Cannabis* yield and quality has resulted in an exponentially increasing interest by the scientific community and the *Cannabis* industry on the study of the effects of LEDs on the plants’ metabolism. The potential of LED lighting in the *Cannabis* sector has been recently reviewed (Lefsrud et al., 2019), in a review that confirms the lack of solid evidence on the effects of light on cannabinoid and terpenoid yields. Treatments of *Cannabis* plants with high-pressure sodium (HPS) and different LED types affected their morphology but had a minor impact on their cannabinoids yields (e.g., CBG, CBD, Δ*^9^*-THC content), as revealed by the GC/FID analyses of the EtOH extracts (Magagnini et al., 2018). However, plants that were grown under LED light had improved Δ*^9^*-THC and CBD concentrations. Additionally, the study concluded that the red to far-red light ratio had no substantial effect on flowering. Based on evidence that was acquired by another study, it has been concluded that different strains exhibiting high Δ*^9^*-THC yield capacity are able to use high levels of photosynthetic photon flux densities (PPFDs). Such observation indicates that the chemovars being tested can be cultivated under high light intensity regimes outdoors or in the greenhouse, under controlled conditions (Chandra et al., 2015).

Nonetheless, although in the literature there is a handful of studies on the effects of environmental parameters (e.g., light, temperature) on the growth of *Cannabis*, there is only a few on the investigation of such effects applying metabolomics. Yet, their impact on *Cannabis* potency and global metabolism is largely unknown. Thus, it is highly expected that the employment of such tool could greatly assist toward the optimization and customization of growth parameters for the production of high quality and standardized products from the various *Cannabis* chemovars.

#### *Cannabis* Plant Protection and Interactions With Biotic and/or Abiotic Factors

Plant pathogenic fungi and pests affect the yield of *Cannabis* cultivations in the greenhouse and outdoors, resulting in devastating quantitative and qualitative losses (McPartland, 1996a, b). Therefore, the optimization of agricultural practices such as foliar or soil applications of registered PPPs (including bioelicitors and biological control agents), that could improve the plants’ productivity and cannabinoid-biosynthetic capacity, and reduce the levels of xenobiotics in the final product (McPartland and McKernan, 2017), are of paramount importance. Such an endeavor could be accomplished through the comprehensive monitoring of plants’ metabolism applying metabolomics, which has a great potential in PPPs’ R&D (Aliferis and Chrysayi-Tokousbalides, 2011; Aliferis and Jabaji, 2011). Nevertheless, *Cannabis* producers, and especially those applying organic farming, currently lack information and guidance on the efficient application of such products (Sandler et al., 2019).

Although the primary MoA for most a.i. of PPPs is known, information on their secondary ones, if non-existent, is largely fragmented (Casida, 2009, 2010; Aliferis and Jabaji, 2011). Although fungicides and insecticides act on functions of the target-organisms that are vital for their survival, they additionally could impact the metabolism of plants (Lydon and Duke, 1989; Garcia et al., 2003; Petit et al., 2012), with the relative knowledge on the undergoing mechanisms being limited. Within this context, the study of the effects of registered PPPs for applications in *Cannabis* cultivation on its metabolism and potency could contribute to the optimization of the agricultural practice (frequency, time of application in relation to the plant’s vegetative stage, dosage) and the selection of the most efficient and safe products.

Of specific interest is the use of phytohormones and PGR, which is a group of PPPs that are integral parts of the agricultural practice for many crops. Phytohormones, in minute amounts, can substantially impact plant processes such as growth, dormancy, and flowering. Abscisic acid (ABA), is a phytohormone that plays a key role as a messenger-molecule by regulating plant responses to biotic and abiotic stimuli, including, among others, salinity, drought, heat, cold, and pathogen infections (Raghavendra et al., 2010). Its application to *Cannabis* at the flowering stage has shown to increase its Δ*^9^*-THC content, however, it causes a decrease in its chlorophyll, steroid, and sterol contents (Mansouri et al., 2009b). Gibberellic acid (GA_3_), another major plant phytohormone, has shown to stimulate the biosynthesis of *Cannabis* terpenoids via the mevalonic acid biosynthetic pathway, but it inhibits the biosynthesis of those that are synthesized via the plastidial methylerythritol phosphate biosynthetic pathway (Mansouri et al., 2009a). Furthermore, in both sexes, GA3 application results in decreased levels of chlorophylls, carotenoids, and Δ*^9^*-THC. The PGR ethephon, which is used in the agricultural practice to regulate plants’ metabolism (e.g., promotion of fruit ripening, flower induction, initiation of reproductive development), has shown to greatly affect the plants’ metabolite composition, including their Δ*^9^*-THC, CBD, and terpenoid content (Mansouri et al., 2016). Although the study was inconclusive on the exact effect of the levels of ethephon on the global *Cannabis* metabolism, it highlighted the potential of this PGR toward the improvement of yield via the regulation of the plant’s metabolism.

PGR are bioactive in very low concentrations and their bioactivity is highly correlated to factors such as the genotypes, the growth stage of the plants and their physiological condition, and environmental factors (e.g., humidity, light, temperature). Therefore, comprehensive studies are further required for the standardization of their applications in *Cannabis* cultivation and the determination of the optimal treatments (e.g., time, doses) under specified environmental conditions, in order to achieve optimum yield and quality.

In addition to the traditional PPPs and biological control agents, the group of endophytes is an alternative source of bioactivity for potential applications in plant protection. They are microorganisms that have developed a mutually beneficial symbiotic relationship with their host, living inside their organism, without causing symptoms (Porras-Alfaro and Bayman, 2011). Numerous *Cannabis* endophytes have been found to compose the *Cannabis* microbiome (Kusari et al., 2013; Scott et al., 2018). Such organisms could be used to increase the resistance of plants to pests and pathogens and possibly in order to modulate the biosynthesis of cannabinoids and other *Cannabis* potent metabolites such as the terpenoids (Gorelick and Bernstein, 2017).

#### Biomarker-Assisted Selection in *Cannabis* Breeding

Through millennia, *Cannabis* cultivation has spread worldwide, resulting in the generation of numerous landrace varieties (strains resulting from human and/or natural selection), and was amongst the first plant species to be domesticated (Clarke and Merlin, 2016; Rahn et al., 2016). The *Cannabis* gene pool has been significantly reduced due to the asexual propagation of strains exhibiting improved yields and potency, inbreeding, the lack of comprehensive germplasm collections (Clarke and Merlin, 2016), and the production of modern strains based on a limited genotypes (Rahn et al., 2016). This, in turn, has additionally resulted in the reduction of its chemical diversity (Mudge et al., 2016, 2018).

Nonetheless, the prohibition of *Cannabis* cultivation and the related research has created a large gap of knowledge on the genetics of the varieties and breeding for desired traits. Currently, a vast number of *Cannabis* strains exist, whose genotypic and metabolic backgrounds are largely unknown. The term “strain” refers to slight phenotypic differences and branding rather than distinct genotypic compositions. The above represent a bottleneck for *Cannabis* R&D toward the development of hybrids exhibiting improved fiber, seed, bioactive molecule-producing capacities, and/or improved resistance to pests and pathogens. There are numerous examples of strains susceptible to pest and pathogen infections, leading to severe yield losses (McPartland et al., 2000; Clarke and Merlin, 2016).

All the above underline the necessity for the comprehensive genetic and metabolic mapping of the existent *Cannabis* strains in order to unravel the relationships between sub-species, the similarities among strains and phenotypes, and to discover single or sets of metabolites-biomarkers that could be further exploited in *Cannabis* breeding programs following biomarker-assisted approaches. Furthermore, the recently introduced legislation on the cultivation of industrial and medicinal *Cannabis* in many countries necessitates the use of certified genetic material from a scientific and industrial perspective.

**FIGURE 7:**
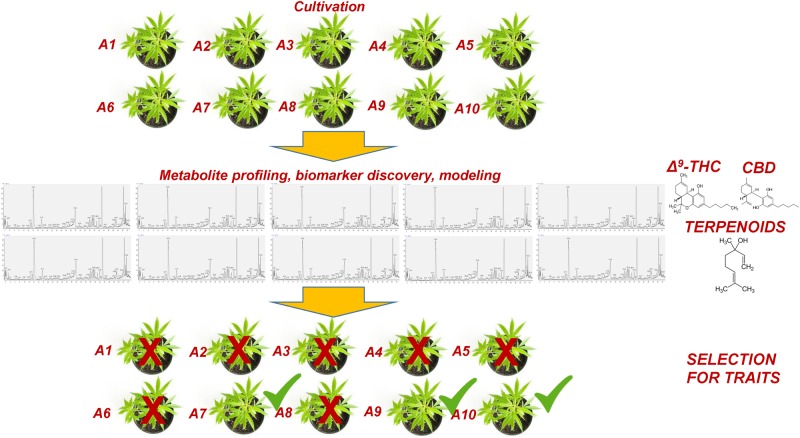
Conceptual pipeline of the biomarker-assisted selection of *Cannabis* (*Cannabis sativa* L.) chemovars based on the desired traits, performing metabolomics

Within this context, metabolomics represents a bioanalytical tool of high potential that could greatly assist and complement the currently applied breeding tools (Taylor et al., 2002; Fernie and Schauer, 2009; Herrmann and Schauer, 2013). This task could be further assisted by the employment of QQQ detectors, which exhibit superior capacities in metabolite quantification and identification (see §3). Although a significant effort has been made toward the improvement of crops via breeding, its capacities in plants’ selection for certain traits have been exploited only recently (Fernie and Schauer, 2009). Being the link between genotypes and phenotypes (Fiehn, 2002; Bino et al., 2004), metabolomics could greatly reduce the required time and the corresponding costs being an integrated component of plant-breeding programs (Figure 7). Focusing on *Cannabis*, its yield, potency, cannabinoid content, flowering, and resistance to pest and pathogen infections, are among the major traits of interest for breeding. In a recent metabolomics study (Mudge et al., 2018), it was shown that *Cannabis* domestication has resulted in an alteration of its metabolism involving the CBDA and THCA biosynthetic pathways. Additionally, the biomarker-assisted breeding could provide insights into attributes such as the “entourage effect,” by breeding for traits related to cannabinoid and terpenoid contents (Grof, 2018).

## Conclusion

*Cannabis* is a species whose exploitation for applications in various fields has sparked great controversy. Nonetheless, there is a consensus that from a scientific perspective, the research on the plant could lead to significant advances for applications of extracts or individual metabolites in medicine, cosmetics, and the food industry. Currently, the recently introduced legislation on *Cannabis* in many countries around the world has enabled research on the plant and the vast array of its products. *Cannabis* matrices are extremely complex, requiring the implementation of advanced bioanalytical tools in order to gain meaningful insights into their bioactivity, medicinal properties, and risk assessment.

Based on its unique capacities and the developments in bioanalytics, is expected that metabolomics will greatly assist in impending *Cannabis* R&D contributing to the development of new, superior, efficient, and safe for the consumer, products. As a functional genomics tool, metabolomics could be ideally employed in the monitoring of cannabinoid and terpenoid profiles and their alterations in response to genotypic changes or agricultural treatments (e.g., fertilizers, bioelicitors, environmental conditions) and also in the biomarker-assisted selection of chemovars.

Additionally, the monitoring and comprehensive mapping of terpenoids could greatly assist the efforts toward understanding their synergy with cannabinoids. The modulation of the potency and medicinal properties of *Cannabis* extracts by their terpenoid content is largely unexplored. The acquisition of information on the effect of terpenoids on the medicinal properties of extracts could accelerate the discovery of novel drugs. The multistep engineering of the terpenoid biosynthetic pathway (Aharoni et al., 2005) and the generation of plants with knock-out mutations via technologies such as the clustered regularly interspaced short palindromic repeats CRISPR (Ran et al., 2013) is feasible (Russo, 2018), and represents a great opportunity. Nonetheless, caution is required in applications of *Cannabis* for commercial purposes, which is expected to spark great controversy and face many regulatory hurdles.

Moreover, metabolomics is an invaluable tool that can be employed in the high-throughput chemotaxonomy or chemotyping of *Cannabis* strains into the corresponding chemovars based on their cannabinoid, terpenoid, and/or global metabolite profiles. Such classification is important not only for research but also for QC purposes. The correlation between *Cannabis* chemovars, their chemical composition, and their medicinal properties, is highly expected to accelerate drug discovery and development. From the current evidence, it is apparent that further experimentation is required for the development of *Cannabis* preparations or individual metabolites as drugs based on clinical trials (Soltesz et al., 2015), for which metabolomics should be an integrated component. Additionally, the employment and integration of advanced analyzers applying metabolomics is strongly expected to provide novel insights toward the understanding of the cannabinoid pharmacokinetics.

The comprehensive study of the effect of light on *Cannabis* metabolism and metabolite profiles could greatly contribute to the deconvolution of the underlying operating mechanisms that regulate the responses of plants to the various light regimes and their potency. This is expected to add a critical mass of information that could be exploited in the optimization of the light conditions in order to regulate its development toward the achievement of, among others, higher yields, improved and customized potency, and early flowering. Furthermore, the research on the scaling-up of the production of rare cannabinoids, cannabis-derived bioactives, or their synthetic analogs through the metabolic engineering of microorganisms, could be substantially accelerated through the application of metabolomics.

Nonetheless, there is a need for further optimization and validation of the available bioanalytical protocols that could be implemented in the routine analyses of *Cannabis* matrices for QC but also for R&D purposes. The robustness of the GC-based platforms, which are the golden standard for metabolomics, faces the challenge of the heat-catalyzed conversions of several cannabinoids, which can be addressed by appropriate silylation protocols. Based on the limitations of the available instrumentation, there is not a single analyzer that could cover the remarkably diverse *Cannabis* metabolome. Additionally, the development of *Cannabis*-specific bioinformatics software and corresponding metabolite databases, would greatly contribute toward the development of metabolomics applications-Canabinomics in *Cannabis*-related research disciplines. To the best of our knowledge, the current is the first overview of the application of metabolomics in *Cannabis* R&D, which following the legalization of medicinal *Cannabis*, is highly foreseen to greatly assist *Cannabis* breeding and selection, being an unparalleled tool to link genotypes with phenotypes and potency, and predict traits based on modeling and machine learning.

## Author Contributions

KA and DB-P conceived the ideas and wrote the manuscript.

## Conflict of Interest

The authors declare that the research was conducted in the absence of any commercial or financial relationships that could be construed as a potential conflict of interest.

The handling Editor declared a shared affiliation, though no other collaboration with one of the authors KA.

## Footnotes

1 https://laws-lois.justice.gc.ca/PDF/C-24.5.pdf

## Abbreviations

Δ*^9^*-THC: Δ *^9^*-tetrahydrocannabinol
CB1, CB2: cannabinoid receptors CB1 CB2
CBD: cannabidiol
GC/FID: gas chromatography-flame ionization detector platform
HRMS: high-resolution mass spectrometry
LC-DAD: liquid chromatography-diode array detector platform
MoA: mode(s)-of-action
NMR spectroscopy: nuclear magnetic resonance spectroscopy
PPPs: plant protection products
QC: quality control
R&D: research and development.
